# Editorial: Collection on renal disease, diabetes and cognitive performance

**DOI:** 10.3389/fnhum.2023.1240691

**Published:** 2023-08-25

**Authors:** Raffaella Franciotti, Stefano Luca Sensi

**Affiliations:** ^1^Department of Neuroscience, Imaging and Clinical Science, G. d'Annunzio University of Chieti-Pescara, Chieti, Italy; ^2^Center for Advanced Studies and Technology (CAST), Institute for Advanced Biomedical Technologies, G. d'Annunzio University of Chieti-Pescara, Chieti, Italy

**Keywords:** kidney disease, diabetes, cognitive impairment, dementia, electroencephalography, functional magnetic resonance imaging (fMRI)

## Introduction

Chronic conditions such as diabetes and renal diseases share neurological comorbidity as both are associated with an acquired and progressive deterioration of cognitive functions (Rayi and Mandalaneni, 2020). Cognitive impairment manifests when one or more of the following domains, like memory, executive functioning, attention, speed of information processing, perceptual motor ability, or language, are targeted (Van Sandwijk et al., 2016). Cognitive dysfunctions could result from many pathological processes that occur inside and outside the Central Nervous System (CNS). Thus, translational studies involving different pathologies could disentangle the mechanisms underlying neural complications.

Although cerebrovascular damage and inflammation might be the common substrates of neurological implications, the neuropathological mechanisms linking diabetes and renal diseases to cognitive impairment are unclear. In addition, the pattern is complicated because kidney disease can result from diabetes.

This Research Topic aimed to address critical aspects of the cognitive decline in patients affected by renal disease and/or diabetes to facilitate knowledge dissemination and establish new trends for future studies.

## Cerebro-renal dysfunction

Chronic kidney disease can affect brain structure and function acutely or chronically at many levels. For end-stage renal disease (ESRD), cerebro-renal dysfunction occurs upon uremic toxin accumulation, cerebrovascular injury, and blood-brain barrier damage (Bobot et al., 2020).

Retention of neurotoxins is associated with neuronal damage, cognitive impairment (Lin et al., 2019), and uremic encephalopathy (Bugnicourt et al., 2013). Encephalopathy caused by the accumulation of uremic toxins is reversible and reduced by dialysis (Viggiano et al., 2020). Thus, this mechanism can be distinguished by neurological conditions characterized by intrinsic degeneration.

Chronic kidney disease enhances levels of cystatin C, pro-inflammatory cytokines, and oxidative stress. Vascular injury causes endothelial dysfunction and acceleration of atherosclerosis, thereby contributing to the development of vascular dementia (Arnold et al., 2016). Neurovascular unit dysfunction can enhance parallel pathogenic pathways as it can alter the clearance of neurotoxic molecules and proteins, such as beta-amyloid (Aβ) and tau protein (Iadecola, 2017)—the pathological hallmarks of Alzheimer's disease (AD).

Both cerebral hypoperfusion and hyperperfusion have been described in patients with chronic kidney disease (Michna et al., 2020). However, hypoperfusion can cause ischemia, and hyperperfusion can disrupt the blood-brain barrier (Nation et al., 2019), causing white matter degeneration and the onset of cerebral microvascular diseases (Mansour et al., 2019), stroke (Shima et al., 2016), epilepsy, and neurodegenerative disorders (Obermeier et al., 2013). Moreover, the increased blood-brain barrier permeability allows the infiltration of noxious agents as cytokines and immune cells, which can contribute to neuroinflammation (Zlokovic, 2008).

Malnutrition-inflammation complex syndrome, a comorbidity of chronic kidney disease (Kalantar-Zadeh et al., 2003), is a possible link between ESRD and neural complications.

## Brain and diabetes

Type 2 diabetes mellitus (T2DM) is characterized by glucose dyshomeostasis, cellular insulin resistance, impaired insulin signaling, metabolic abnormalities, and chronic inflammation. The disruptive effects of T2DM on the brain are well-recognized. T2Dm is a major risk factor for cognitive decline and dementia (Verdile et al., 2015), particularly AD.

Chronic hyperglycemia accelerates the accumulation of extracellular Aβ aggregates and intracellular hyper-phosphorylated tau protein and drives and/or enhances inflammatory and oxidative stress processes that promote neurodegeneration (Bharadwaj et al., 2017). Cell culture and animal studies have indicated that the early accumulation of Aβ may play a role in CNS insulin resistance and impaired insulin signaling, thereby linking T2DM to neurodegeneration.

T2DM can also produce cerebrovascular damage and further vascular-dependent cognitive impairment (Luchsinger, 2012). Indeed, the presence of cerebral infarcts (Saczynski et al., 2009) and markers of microvascular disease (Moran et al., 2013) predict an increased risk of future dementia in T2DM. The involvement of the vascular pathway in the appearance of dementia in T2DM patients is debated. Studies reported conflicting results (van Elderen et al., 2010; Qiu et al., 2014), and whether T2DM can cause cognitive impairment due to neurodegeneration or vascular cognitive impairment remains to be elucidated.

## Key aspects of cognitive decline in renal disease and diabetes

In this Research Topic, five papers investigated the neural substrate of cognitive decline in patients with renal disease or T2DM, using neuroimaging techniques (i.e., electroencephalography (EEG) and functional magnetic resonance imaging) and one paper (Higgins Tejera et al.) evidenced that the association between the cystatin C level and dementia prevalence could be increased by racial and ethnic disparities.

The Systematic Review and meta-analysis by Cao et al. on 11 resting-state functional magnetic resonance imaging (rs-fMRI) studies showed that ESRD patients with cognitive impairment exhibit decreased resting-state activity compared to healthy controls. In brain areas that included the default mode, visual recognition, and executive control network, compared to controls, the amplitude of the low-frequency fluctuations was lower in patients.

Zhang et al. evaluated the brain networks' topographic organization and efficiency of ESRD patients with and without cognitive impairment and undergoing maintenance hemodialysis. Whereas, the efficiency of the global information processing -measured by small-worldness indices- was similarly altered in both patients' groups, local efficiency -measured by the clustering coefficient- significantly correlated with different degrees of cognitive dysfunction.

The paper of Jatupornpoonsub et al. (a) found altered EEG patterns in ESRD patients with the malnutrition-inflammation complex syndrome. Cognitive dysfunction, motor, and psychiatric disturbances correlated with the absolute power and coherence in delta-theta bands and absolute power and amplitude asymmetry in the beta 1 band. These EEG abnormalities differ from those found in patients with neurodegenerative processes (Lizio et al., 2018).

Enhanced neurological complications due to the co-presence of renal disease and diabetes were investigated by Jatupornpoonsub et al. (b) using resting-state EEG recordings. The authors found that the power ratio between delta and theta bands in fronto-parietal areas was lower in patients than in healthy controls and lower in patients with co-existence of renal disease and diabetes than in patients with renal disease. The power ratio between theta and beta bands was higher in patients with both diseases than in patients with renal disease. The reduced delta and beta activity in patients with both renal disease and diabetes could be related to encephalopathy and attention deficits, supporting the hypothesis of reduced cognitive processing in patients with both renal disease and diabetes.

The neural basis of cognitive impairment in patients with diabetes was also explored in the study of Lei et al.. The authors found an abnormal positive correlation between the activation of the dorsal attention network and the default mode network in T2DM patients. They propose that this abnormal interaction may be the neural basis of altered attention and general cognitive dysfunctions in T2DM.

## Perspectives

In conclusion, this Research Topic provided supporting findings on the cerebral alterations of the patients affected by kidney disease and/or diabetes. Results were focused on the attention control system, as attention is the initial, primary stage for the generation and performance of complex cognitive processing. Future multidisciplinary studies should disentangle mechanisms that link cognitive impairment to these chronic diseases, implement preventive strategies and generate early-focused therapeutic interventions.

## Author contributions

All authors listed have made a substantial, direct, and intellectual contribution to the work and approved it for publication.
